# Research Engagement in Canadian Anesthesiology Residency: A Survey Study

**DOI:** 10.7759/cureus.115241

**Published:** 2026-08-26

**Authors:** McKenna Postles, Anna Ratcliffe, Nicholas West, William Yao, Alexa Caldwell, Zoe Brown

**Affiliations:** 1 Faculty of Medicine, University of British Columbia, Vancouver, CAN; 2 Pediatric Anesthesia Research Team, BC Children's Hospital Research Institute, Vancouver, CAN; 3 BC Children’s Hospital Pediatric Anesthesia Fellowship Program, Department of Anesthesiology, Pharmacology and Therapeutics, University of British Columbia, Vancouver, CAN; 4 School of Anaesthesia, Peninsula Postgraduate Medical Education, Plymouth, GBR; 5 Anesthesiology Residency Program, Department of Anesthesiology, Pharmacology and Therapeutics, University of British Columbia, Vancouver, CAN; 6 Department of Anesthesiology, Pharmacology and Therapeutics, University of British Columbia, Vancouver, CAN; 7 Department of Anesthesia, BC Children's Hospital, Vancouver, CAN

**Keywords:** anesthesiology resident, medical residency, medical resident education, research engagement, resident engagement

## Abstract

Introduction

This study investigated Canadian anesthesia residents’ research participation and expectations, identified barriers and enablers, and the need for a Canadian trainee-led research network (TRN).

Methods

With Research Ethics Board approval, this study invited residents from all Canadian training programs, approximately 700 residents nationally, to complete an anonymous online survey. The survey included sections on prior research experience, expectations of research during residency, academic career aspirations, scholarly output during residency, barriers and enablers to engaging with research, and opinion regarding the value of a TRN.

Results

Eighty-two participants responded from fourteen training programs: 69 (84%) completed all questions and were included in the analysis. Forty-nine (71%) had been involved in research during residency. Most felt their faculty provided ample research support (n = 44, 64%; 95% CI, 51% to 75%, p = 0.030), but many expressed a lack of research interest (n = 44, 64%; 95% CI, 51% to 75%, p = 0.030). Participants would value a TRN that provided access to mentorship (n = 47, 68%; 95% CI, 56% to 79%, p = 0.004) and disseminated information about opportunities (n = 46, 67%; 95% CI, 54% to 77%, p = 0.008).

Conclusion

Despite faculty support, many residents lacked the interest to engage in research. In other countries, TRNs have been successful in involving residents in high-quality, multi-site research. Our findings support a recommendation that Canadian anesthesiology programs should consider a similar approach to inspire and facilitate residents’ involvement in research.

## Introduction

Involvement in medical research encourages the practice of evidence-based medicine, leading to improved patient outcomes [1]. Anesthesiology residency programs must teach clinical skills, but also equip trainees with the skills for active involvement in research, critical appraisal of evidence, and the generation and application of new knowledge [2]. The Royal College of Physicians and Surgeons of Canada requires that anesthesiology residency programs provide residents with the opportunity, access, and mentorship to undertake scholarly activities [3,4].

International and national studies [5-12] have identified constraints across multiple disciplines, including a lack of mentorship, interest, resources, and dedicated time to undertake research. A 2015 Canadian study reported that 69% of residents had participated in research during residency and a third of anesthesiology residents were interested in undertaking research during residency and in their future career [13]. Current research on Canadian anesthesiology resident research outputs and perspectives is limited.

In the UK, multiple medical specialties, including anesthesia, have established trainee-led research networks (TRNs). Recently, 15% of the UK’s anesthetic trainees (over 750 individuals) took part in a resident-led national prospective multicenter observational study to measure post-operative pain and pain relief [14]. This exemplifies how TRNs can facilitate trainee collaboration under the mentorship of academic physicians across multiple sites to deliver high-quality collaborative multicenter research. Collaborative research models in the UK, Australia, and New Zealand have delivered multicenter observational research involving as many as 400 to 1350 local collaborators in national projects [14-16] and even more in international projects [17], as well as national audit projects [18] and regional or national surveys of practice [19].

Hence, this study aimed to investigate Canadian anesthesia residents’ current involvement in and expectations of research, and to identify key barriers and enablers to engagement. Secondly, we aimed to use these responses to identify requirements for developing a proposal for a Canadian TRN model.

## Materials and methods

Study design

This cross-sectional survey study was approved by the University of British Columbia (UBC) Children’s and Women’s Research Ethics Board (H23-03889) and was conducted between January 2024 and March 2024. Participants were current residents invited from all years and all anesthesiology residency programs across Canada; anesthesiology fellows and non-anesthesia trainees were excluded. This manuscript follows the Consensus-Based Checklist for Reporting of Survey Studies (CROSS) consensus guidelines for reporting survey-based research [20].

Survey design and distribution

The survey was developed by a multi-disciplinary group, including: a pediatric anesthesiology fellow contributing experience of TRNs in the UK; anesthesiology resident research leads for the UBC anesthesiology residency program, bringing lived experience of the Canadian training program and resident perspectives; the UBC anesthesiology program director, bringing system-level perspectives; and methodology support from the BC Children’s Hospital Pediatric Anesthesia Research Team. The survey was created on Research Electronic Data Capture (REDCap) hosted at BC Children’s Hospital Research Institute [21].

The survey was designed to take 10-15 minutes and included up to 42 questions within four sections: A) demographics (residency program, year, gender); B) research before and during residency (prior research experience, expectations of research during residency, academic career aspirations, scholarly output during residency); C) barriers and enablers to undertaking research in residency; D) research network (opinions regarding the value of a TRN). Multiple-choice and five-point Likert-style questions (strongly disagree to strongly agree) were used to assess participants’ current research expectations, involvement, and barriers and enablers to research engagement. The lists of potential barriers and enablers were identified using previous literature and experiences of the author group [5-7,9-12], subsequently refined and reduced in number through group discussion, and worded to fit the Canadian training environment. Likert-style questions (strongly disagree to strongly agree) assessing participants’ opinions of a Canadian TRN model were used in conjunction with primary outcome data to inform the secondary outcome of identifying requirements for developing a Canadian TRN model. One open-ended free-text question at the end of the survey invited participants to provide further feedback and comments. Five UBC anesthesiology residents independently pre-tested the questionnaire on REDCap to provide feedback on wording, ambiguity, clinical sensibility, and timing.

A REDCap auto-generated online survey link was emailed to the program directors of the 17 Canadian anesthesiology residency programs on January 19, 2024, to be distributed to their residents in the third week of January 2024, with a reminder to residents sent one week later. Consent was implied by completing the survey, and eligibility was verified in Section (A): Demographics, where participants were asked for their anesthesia residency year and location. Participants could complete the survey on their mobile device or a computer, and their responses were stored in the REDCap database. The survey was distributed to residents as an anonymous survey link, so it was not possible to track missing or duplicate participation. On completion of the survey, residents were offered the chance to enter a prize draw to win one of seventeen gift cards. The survey was closed on March 19, 2024.

Data analysis

Sample Size

We estimated approximately 700 potential participants from 17 programs nationally. Only participants who responded to all survey questions were included in the analysis.

Statistical Analysis

Quantitative results were analyzed using Excel (Microsoft® Corp., Redmond, WA), tabulated, and reported as counts with percentages. Demographics were reported by residency location based on Canadian region. Five-point Likert-style responses were displayed graphically to visualize the distribution of responses for each question. The 95% confidence interval (CI) for the proportions of respondents agreeing/disagreeing with each statement was calculated using R version 4.4.0 (R Foundation for Statistical Computing, Vienna, Austria) to identify significant results.

## Results

Eighty-two participants responded; 69 (84%) completed all questions and were included in the analysis, with an estimated response rate of 10%. Respondents were from every postgraduate year (PGY) of residency and from all programs (Atlantic Region: n = 13; Central Canada: n = 22; Prairie Provinces: n = 22; West Coast: n = 19), except Université de Sherbrooke, Université de Montréal, and Université Laval. Thirty-seven participants (54%) were female; 16 (23%) had completed a master’s degree before residency.

Forty-nine (71%) residents had been involved in one or more research activities during their residency, including quality improvement (n = 24, 35%), clinical research (n = 16, 23%), educational projects (n = 6, 9%), and basic science research (n = 3, 4%). Residents had disseminated research results through conference presentations (n = 23, 33%) and/or publications (n = 11, 16%; Table 1).

**Table 1:** Canadian anesthesiology residents’ current research output summary by residency year. ^*^Participants (n = 69) were asked, “During your anesthesiology residency program, have you achieved any of the following?” Participants were allowed to click more than one achievement (e.g., select both publication and presentation) and more than one sub-achievement (e.g., select both data collection and data analysis). ^†^Study design and development included options for idea development, study design, protocol writing, and ethics application. Respondents were counted if they selected any of these options.

Research output^*^	Residency year					Total (N = 69)
	PGY-1 (n = 13)	PGY-2 (n = 15)	PGY-3 (n = 14)	PGY-4 (n = 16)	PGY-5 (n = 11)	
Publication: First or co-author for original research or other project	2 (15%)	0	4 (29%)	5 (31%)	0	11 (16%)
Presentation: International, national, regional or local oral or poster presentation	1 (8%)	1 (7%)	6 (43%)	8 (50%)	7 (64%)	23 (33%)
Basic science research: Idea development, funding application, experimental work, or write-up/publication	0	0	1 (7%)	2 (13%)	0	3 (4%)
Clinical research:	0	4 (27%)	5 (36%)	5 (31%)	2 (18%)	16 (23%)
Single-site research project	0	4	5	4	2	15
Multi-site research project	0	0	0	1	0	1
Study design and development^†^	0	2	4	4	1	11
Patient recruitment	0	3	2	3	1	9
Data collection	0	3	3	4	2	12
Data analysis	0	3	3	3	2	11
Presentation of results	0	2	3	3	2	10
Write-up/publication	0	2	4	3	0	9
Quality improvement: Undertaken project or presented results	2 (15%)	6 (38%)	5 (36%)	3 (19%)	8 (73%)	24 (35%)
Educational project: Simulation, curriculum development, design of course, or delivery of lecture	0	1 (7%)	1 (7%)	3 (19%)	1 (9%)	6 (9%)
None	9 (69%)	5 (33%)	3 (21%)	2 (13%)	1 (9%)	20 (30%)

**Figure 1:**
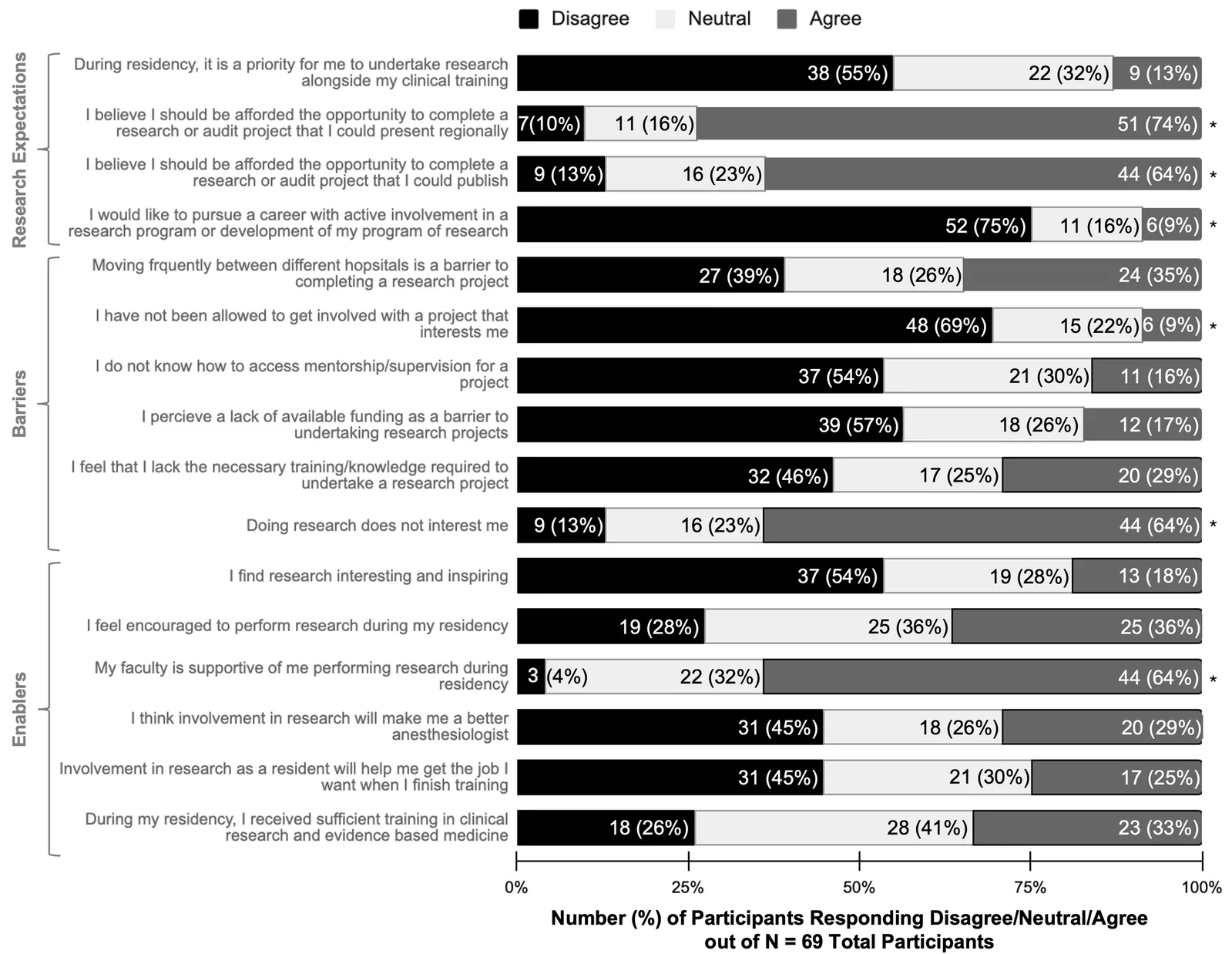
Canadian anesthesiology residents’ expectations and perceived barriers and enablers to research during residency, ranked as strongly disagree, disagree, neutral, agree, or strongly agree and grouped into three groups per statement (disagree, neutral, agree). ^*^Clear agreement or disagreement with the statement (p < 0.05 and 95% confidence interval does not include 50%).

Many participants agreed that they should be provided research opportunities to present regionally (n = 51, 74%; 95% CI, 62% to 83%, p < 0.001) or to publish (n = 44, 64%; 95% CI, 51% to 75%, p = 0.030) during residency, although 52 (75%, 95%CI 63% to 85%, p < 0.001) said they did not want to remain involved in research during their careers (Figure 1).

The most agreed-upon barrier to research was a lack of interest (n = 44, 64%; 95% CI, 51% to 75%, p = 0.030), although participants generally agreed that they had been allowed to get involved in a project that interested them (n = 48, 70%; 95% CI, 57% to 80%, p = 0.002; Figure 1). The most agreed-upon enabler for research was receiving ample faculty support (n = 44, 64%; 95% CI, 51% to 75%, p = 0.030) rather than the provision of protected time, resources, interest and inspiration, and incentives to further their career (Figure 1).

Participants would value a research network that gave them access to academic mentorship (n = 47, 68%; 95% CI, 56% to 79%, p = 0.004), facilitated engagement of residents with research through disseminating information about upcoming opportunities (n = 46, 67%; 95% CI, 54% to 77%, p = 0.008), and allowed them to work collaboratively with other residents to deliver multi-site research (n = 42, 61%; 95% CI, 48% to 72%, p = 0.092).

Free-text comments (n = 9) expressed an interest in broadening the definition of research projects: for example, one participant (PGY-3) “strongly encourage(d) broadening the definition of scholarly or research work for the sake of completing academic projects in residency.” They stated that for those “who have done advanced graduate degrees, we know how intensive research can be, and the focus and protected time it takes to do it well. This is not feasible in residency, so rather than doing a watered-down minor research project, it would be great to explore other types of academic work that may be new in residency and very valuable, such as curriculum development, teaching, simulation, residency training expansion/ network building, community clinical outreach, and quality improvement implementation.”

## Discussion

This survey of research experience and engagement among Canadian anesthesiology residents found that 71% of respondents had been involved in at least one research activity, aligning with a previous survey, which reported that 69% of Canadian anesthesiology residents had participated in research [13]. Most residents believed they should be provided with the opportunity to present or publish during their residency. Respondents, who included trainees from all residency years across all regions of Canada outside Québec, reported a lack of interest as the key barrier to engaging with research, despite supportive faculty and programs. Although most respondents had been given research opportunities during their training, their identifying lack of interest as a barrier and their disinterest in pursuing future research are troubling. These findings contrast with research indicating that lack of time, insufficient skills, and lack of mentorship are the most common barriers to research participation among trainees [22].

Despite a lack of interest, participants agreed that they should be provided the opportunity to do research in residency and would value a TRN that would provide mentorship and share information about research opportunities. It is possible that despite faculty support, the types of research projects available to residents are uninspiring or lack feasibility alongside their clinical training. Currently, resident attitudes toward research are in discordance with the physician competencies taught in their training programs [2,23]. The implementation of a Canadian TRN could improve engagement; in the UK, the TRN model has been used to generate study ideas that trainees find relevant and interesting [24] and has provided opportunities to take on leadership roles, which has been found to be an important motivator for engaging in trainee-led research [25]. Additionally, TRN implementation would provide increased access to academic mentorship and collaborative networking opportunities by facilitating multi-site research with students and faculty in other regions, which most residents indicated would be valuable [17,26]. A TRN could also facilitate respondent suggestions to broaden the research project requirement to inspire residents to gain more experience in their areas of interest.

In the UK, TRNs take advantage of a centralized ethics application, allowing for collaborative research across multiple regions [22]. The lack of a single-site ethics application may limit the implementation of a Canadian TRN, but local faculty mentorship and training should support residents through these processes. Alternatively, collaborative research projects could involve a large number of residents in a long-term single-site study, allowing them to transiently engage with the project during their rotation. UK TRNs are also very well supported by national policymakers, including the Royal College of Anaesthetists, which requires trainees to engage with TRNs as a part of their training curriculum [27,28], and initiatives such as the Sprint National Audit Projects (SNAP studies) [29], and the National Institute for Academic Anaesthesia, which actively aims to develop and promote trainee involvement with research. There are similar systems in the UK to support surgical TRNs [30]. Canada may also benefit from this approach.

There are limitations to our study. Firstly, we did not translate our survey into French and received no responses from training programs in Québec. This was a practical constraint at the time of implementation, but it clearly limits the generalizability of our findings and should be addressed in any follow-up or discussion about training initiatives that might arise from this work. Our overall response rate was low (approximately 10%), but all other anesthesia training programs, except those in Québec, are represented, with one or more respondents from each training year across each of the Atlantic Region, Central Canada, Prairie Provinces, and West Coast. Secondly, as survey responses were anonymous, we were not able to track responses against invitations sent or received. The survey was sent out by program directors using institutional distribution lists, and we cannot report the number of residents to whom the invitation was sent, how many received it, how many were missing from those lists, or if/when reminders were sent. These uncertainties should be considered when interpreting our results. Thirdly, the survey was designed, built, and tested by a team from a single West Coast training program, and we did not engage other programs to contribute questions or test the survey.

## Conclusions

Our findings support the recommendation that Canadian anesthesiology programs consider a similar approach to inspire residents and future anesthesiologists to participate in research. Future work should include: translation of this survey to French and redistribution to those programs that did not respond; a more focused survey of residents to determine feasible project ideas that interest them; and an initial pilot study at a few sites in BC, inviting residents to suggest project ideas, with analysis of feedback and engagement.

With senior academic support and mentorship, collaboration between large numbers of trainees can deliver impactful research projects alongside training and high-intensity clinical work. Those who participate in these projects gain the satisfaction of seeing the results of their work published and influencing practice. It is hoped that making research more accessible, inspiring, and rewarding to residents could spark an interest in research throughout their career. It would take enthusiasm from a few residents and staff, but with imagination, social media, and instant messaging, it has potential.
